# Becoming virtual: a preliminary experience of outpatient primary care during COVID-19 pandemic

**DOI:** 10.11604/pamj.2020.37.262.26574

**Published:** 2020-11-24

**Authors:** Syed Irfan Karim, Farhana Irfan, Mohammed Ali Batais

**Affiliations:** 1 Department of Family and Community Medicine, College of Medicine, King Saud University, Riyadh, Saudi Arabia,; 2 Medical Education Research and Development, Department of Family and Community Medicine, College of Medicine, King Saud University, Riyadh, Saudi Arabia

**Keywords:** Coronavirus, COVID-19, primary health care, pandemic

## Abstract

The World Health Organization (WHO) has declared COVID-19 outbreak as a pandemic. This pandemic is transforming the world and has posed exceptional challenges to health care delivery. Saudi Arabia has exerted unprecedented efforts and measures to fight the pandemic. Appreciating the value of primary health care during this crisis the family and community medicine department reorganized the services. We discuss the problems faced, solutions and lessons learned in the hope others may find it helpful.

## Commentary

The World Health Organization (WHO) has declared COVID-19 outbreak as a pandemic [1]. COVID-19 has spread globally; from East to West and this pandemic has posed exceptional challenges to health care delivery [2]. Among the Muslim countries, Saudi Arabia is the holiest city in the Islamic world and particularly at high risk due to its status and pilgrims attending the holy cities from all over the world. As a part of the world which exerts unprecedented efforts and measures to fight the pandemic; the ministry of health in the Kingdom of Saudi Arabia has taken strict precautionary measures by suspending religious activities, border control; testing and quarantine of individuals with history of contacts and at home self- isolation. As of June 2020, the Kingdom had 348,037 confirmed cases including 21,284 active cases and only 5437 tallies of fatalities [3]. Despite the unlimited support from the state to fight the pandemic, the country and the medical system is facing problems due to the growing COVID-19 caseload. An important measure taken by the Ministry of Health was recognizing the value of a strong coordinated response to this crisis from a primary health view and implementing the best practices that are conductible across the country and in the region. This was essential as primary health care clinics are the first point of contact in the health system for most cases. The information on management strategies of COVID-19 in the primary health care setting is inadequate in the region. This highlights the pressing need to enhance our understanding of the response of primary care settings during this COVID 19 crisis. We describe our strategies to inform others who are making this transition of how primary care clinics were transformed to respond to this pandemic.

**Figure 1:**
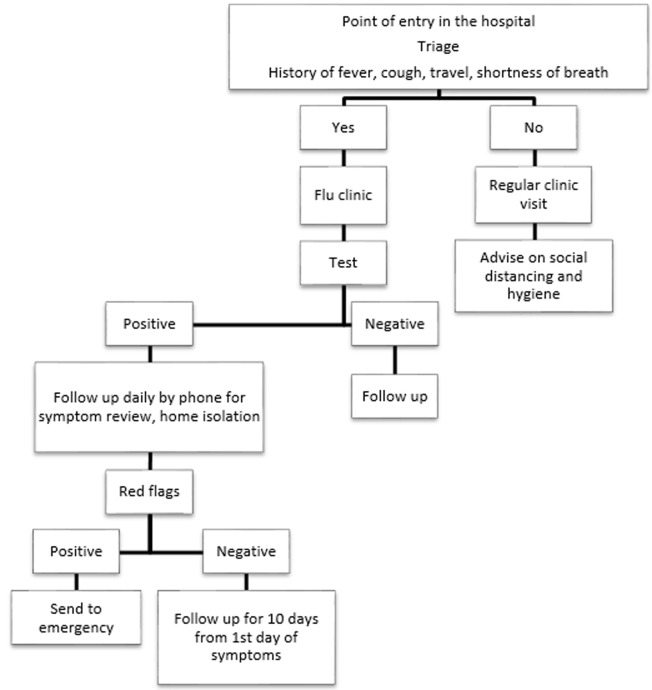
institution workflow for patients with respiratory tract symptoms

**What problems were addressed:** we describe our strategies implemented at the primary care clinics in one of the biggest medical facility King Saud Medical City (KSUMC) in the capital city; Riyadh. The primary care services at KSUMC were urgently modified in March 2020 based on the four key pillars: surveillance and detection, clinical management, prevention of the spread and maintaining essential services. The department of Family and Community Medicine developed a system for remote assessment and treatment of patients and introduced WhatsApp clinic service along with virtual clinics for medical inquiries to be solved. The aim was to continue virtually the disease monitoring and checkups along with the screening activity. The practice staff were required to work remotely and access to clinical systems was given to allow the day-to-day operation to continue. For walk in patients, strict infection control measures were taken and the patients were streamlined according to the hospital protocol (Figure 1). Wearing a mask was compulsory and screening quaternaries were obtained regarding COVID-19. All medical staff were trained in donning and doffing of personal protective equipment (PPE). The health care workers (HCW) were trained and certified by the infection control department.

**What did we do?** the primary care services implemented the changes in the first week of March 2020. As a first step, physical distancing by developing a system for remote assessment and treatment of patients from in- person care to telephone consultation in just a few days. In speedily adopting this system, our goal was the safety of patients and our front line health care workers, as well as addressing their concerns and anxiety about personal safety [4]. As a direct response to COVID-19, practice staff were required to work distantly. A drive to improve the footfall in individual clinics, keeping in mind the safety. Patients were advised to contact through WhatsApp by sending their file number and stating their medical inquiry, which was answered by the relevant WhatsApp support group. In virtual practice, telephone care was the telemedicine mainstay. Most of the physicians provided care from their homes or office space. As KSUMC follows practice based on lists of registered patients, with knowledge of patients´ demographics and co-morbidities; the appointment types were shifted from face to face consultation to phone and virtual visits. In addition, new modes of communication were incorporated like websites, portal messages and social media. The Family Medicine department with the support of the Information Technology (IT) department developed a Virtual Clinic website, by the name of OSRATY platform; a secure online website that gives 24-hour access. The system worked for physicians by providing secure access to patient records, ability to view list of patients for upcoming weeks, their preference for direct call or virtual call, direct message facility to invite patients and view patients self- reported COVID 19 score before consultation by using a secure username and password through VPN. It worked for patients by sending a unique patient appointment link through SMS. Patients could access the clinic by smart phone or desktop and have live consultations with their family physician. The pharmacies also began home deliveries or curbside pickups. The net effect was reduced exposure between patients and staff.

**Containment and mitigation strategies:** to minimize the risk of transmission from infected to non-infected individuals' patients were contacted by the physicians and reviewed via telephone call. Although seeing is a vital tool for any physician [5], physicians used their professional judgement in most of the consultations to decide about patient health needs. In the actual process of a consultation we followed the principals of a telephone consultation and if the patient informed about any flu symptoms like fever, cough, sore throat or a travel history, they were advised keeping in mind the natural progression of flu and followed up for 3-5 days. They were given instructions on home isolation, social distancing and improved personal and environmental hygiene along with safety netting and how to get the best care available. Guidance and Sign posting were done for the online resources and apps that were introduced during COVID pandemic by the Ministry of Health (MOH) and sites that were evidence based and updated. Patients with persistent symptoms were advised to visit the flu clinic for further workup while those who had red flag signs were directed to emergency department for further evaluation. For safety and legal issues, calls were documented. Routine patients who were stable were given next routine appointment (Figure 1). After implementation of the new system, from 18 March 2020 to August 2020, a total of 10230 messages were received via WhatsApp. Medical inquiries 43% (n=4398) refill of medications 23% (n=2352), appointment 22.9% (n= 2352) and lab results 11% (n= 1125). Out of 23798 patient´s consultations at the three primary care clinics after implementation of the new system, a total of 15061 patients (91%) were booked while 8737 patients (9%) were walk in. Out of the booked patients, virtual phone consultations were done for a total of 12990 (86%) patients and 2071 (14%) patients did not reply.

**What problems were faced and their solutions?** From the patient’s perspective, the virtual consultation offered safe management for many, which was convenient, timely and quick option for seeing the physician. However, there were some drawbacks in calling the patients, due to the fact that many patients would be asleep and hence not attend calls in the morning hours, also all patients were not technologically savvy or elderly who had hearing impairment. Initially patients were not familiar with the new system and were overwhelmed. From the staff perspective, there were problems with easy approachability to the resources. Initially, the physicians faced problems in accessing the system from home. Other challenges encountered were managing the safety of staff, lack of full personal protective equipment endangering staff health and managing patient demands. Challenges with a lower impact were increased overall workload due to no medical students and residents allowed on the clinics, medical student and registrar placements, technology infrastructure, staff working from home or self-isolating.

**What lessons were learned?** virtual clinics is a useful experience when real patient contact is not possible. It enables physicians and patients to share information, converse at their convenient time 24/7, using their phones or computers. In-person visits might become not a preferable mode or option for meeting patient needs in future as patients prefer convenient and economical care [6]. King Saud University Medical city (KSUMC) eSahi tele-medical systems have been successfully deployed to evaluate and treat patients without referring them to in-person care. However, unfortunately the OSRATY platform was not completely functional and few services were not available. Clinicians were able to improvise with patients for telemedicine consultation. We were surprised by the highly interactive sessions achieved by utilizing this modality. Educating patients on web-side ensured a pleasant experience for all those involved. Regular quality assurance checks on the services are needed to identify any risks and failures. Telehealth is an addition to the in- person consultation format and addition of video calls may be a better option. Though it is not ideal, it´s well suited for the present scenario where clinicians are able to see patients. Telemedicine consultation might become a necessary expertise for the next generation of physicians both inside and outside a pandemic [7].

**Our future vision:** the primary care in Saudi Arabia has made a valuable contribution to the population in managing COVID-19, preventing other diseases and providing continuous care to those with existing health conditions. The primary health care services are working to provide a different kind of care experience by changing the health care delivery system. The aim is to make the process of telemedicine smoother and overcome the societal and technological barriers. With the increasing support of the administration, we hope to implement all the OSRATY services and make it viable for the public in near future.
